# Contrações Atriais Prematuras Originadas da Veia Cava Inferior: Uma Fonte Arritmogênica Rara Passível de Ablação

**DOI:** 10.36660/abc.20250555

**Published:** 2026-05-11

**Authors:** Ningning Zheng, Yiyuan Chen, Feng Xue, Fangfang Zhang, Lin Ling, Tingbo Jiang

**Affiliations:** 1 The First Affiliated Hospital of Soochow University Department of Cardiology Suzhou China Department of Cardiology, The First Affiliated Hospital of Soochow University, Suzhou City, Jiangsu Province – China

**Keywords:** Complexos Atriais Prematuros, Veia Cava Inferior, Ablação por Radiofrequência

## Introdução

As contrações atriais prematuras (CAPs) são arritmias cardíacas comuns, tipicamente associadas a mecanismos desencadeadores. CAPs originadas nas veias pulmonares (VPs) são consideradas importantes desencadeadores de fibrilação atrial (FA), e o isolamento das veias pulmonares (IVP) tem sido amplamente utilizado como tratamento.¹ No entanto, a veia cava inferior (VCI) é geralmente considerada uma região eletricamente quiescente, e estudos sobre descargas ectópicas originadas na VCI são relativamente raros. Neste estudo, apresentamos um caso de CAPs originadas na VCI, com o objetivo de fornecer novas informações sobre os mecanismos fisiopatológicos dessas arritmias.

## Apresentação do estudo

### Características do paciente

Uma paciente de 78 anos apresentou palpitações recorrentes, aperto no peito e fadiga nos últimos 9 meses. Cada episódio dura de 10 a 30 minutos e ocorre aproximadamente 5 a 6 vezes por mês. Ela negou sentir dor ou desconforto no peito. Vários eletrocardiogramas (ECGs) mostraram CAPs. A paciente havia sido tratada com um betabloqueador, mas não houve melhora significativa dos sintomas e, consequentemente, ela foi encaminhada ao nosso hospital.

**Figura 1:**
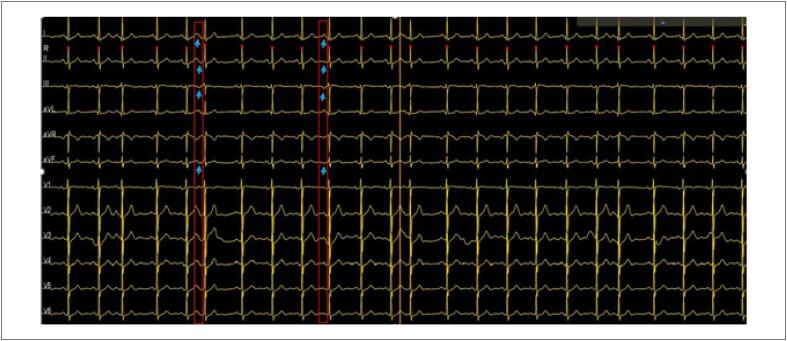
Eletrocardiograma de 12 derivações mostrando contrações atriais prematuras com características morfológicas típicas.

O ecocardiograma transtorácico (ETT) revelou discreto aumento do átrio esquerdo, com diâmetro atrial esquerdo de 40 mm. A angiotomografia computadorizada coronária mostrou uma placa não calcificada no segmento proximal da artéria coronária direita, acompanhada de estenose luminal leve. Os demais resultados dos exames laboratoriais estavam dentro dos limites normais. Durante a hospitalização, um ECG de 12 derivações revelou CAPs frequentes com morfologia de onda P negativa nas derivações inferiores (II, III e aVF) e morfologia de onda P positiva em V1 (Figura 1). O monitoramento Holter revelou CAPs frequentes (15.838 eventos em 24 horas). A paciente não tinha histórico de cirurgia cardíaca ou ablação por cateter. Ela foi diagnosticada com CAPs frequentes e totalmente informada sobre o estado da doença e os potenciais benefícios e riscos das diversas opções de tratamento. Ela optou por se submeter a estudo eletrofisiológico (EEF) e ablação por radiofrequência (ARF).

### Procedimento de ablação

A ecocardiografia transesofágica (ETE) pré-procedimento confirmou a ausência de trombo no apêndice atrial esquerdo. Sob anestesia local, um cateter decapolar foi posicionado no seio coronário (SC) e um sistema de mapeamento eletroanatômico tridimensional (CARTO 3, Biosense Webster, CA, EUA) foi utilizado para reconstruir o átrio direito (AD), o septo interatrial e os óstios das veias cavas, e para realizar o mapeamento de ativação. O mapeamento de alta densidade foi realizado com um cateter diagnóstico multipolar (Pentaray^®^ Nav Eco, Biosense Webster). A ARF foi realizada utilizando um cateter com irrigação aberta e sensor de força de contato (THERMOCOOL SMARTTOUCH SF, Biosense Webster, Diamond Bar, CA, EUA) com as seguintes configurações: 40 W; irrigação 17 mL/min; duração da lesão de 20 a 30 s por aplicação.

O EEF e o mapa de ativação inicial indicaram que as CAPs clínicas se originavam da veia pulmonar inferior direita; portanto, o IVP foi realizado. Apesar do isolamento elétrico confirmado de todas as quatro VP, as CAPs persistiram. O mapeamento de ativação subsequente localizou a ativação atrial mais precoce na região próxima à fossa oval. A ablação nesse local não reduziu materialmente a carga de CAPs.

**Figure 2:**
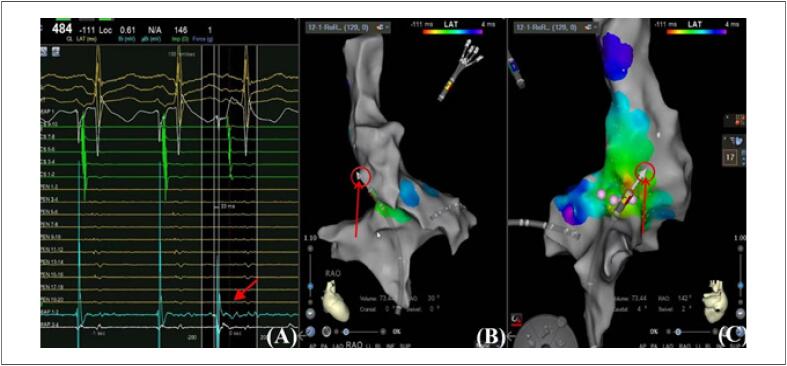
A) Uma onda na parede posterior inferior do átrio direito, próxima ao óstio da veia cava inferior, foi precedida por 33 ms no eletrocardiograma intracardíaco. B e C) Mapas tridimensionais de ativação focal do átrio direito e dos óstios da veia cava; o círculo vermelho e a seta indicam o local da ablação por radiofrequência na junção VCI-AD.

Dada a potencial associação entre CAPs e áreas de baixa voltagem no AD, foi realizado mapeamento de voltagem no AD, que não revelou áreas discretas de baixa voltagem. Isoproterenol intravenoso (8 μg/min) foi administrado para atingir uma frequência cardíaca >100 bpm e provocar CAPs estáveis, seguido de novo mapeamento. O mapeamento de alta densidade localizou a ativação mais precoce na parede póstero-inferior do átrio direito, próximo à junção da veia cava inferior com o átrio direito (VCI-AD), onde o eletrograma atrial local precedeu o início da onda P de superfície em 33 ms (Figura 2).

**Figura 3:**
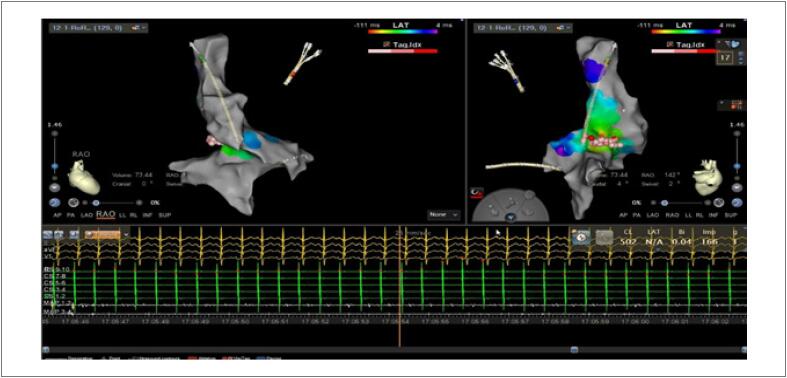
Ausência de CAPs após ablação na veia cava inferior no eletrocardiograma intracardíaco.

O mapeamento por estimulação foi subsequentemente realizado no local de ativação mais precoce, e a morfologia da onda P estimulada correspondeu de perto às CAPs espontâneas em todas as 12 derivações do ECG de superfície. Simultaneamente, o bipolo CS 9-10 no cateter do seio coronário registrou continuamente CAPs correspondentes à morfologia clínica. Após a exclusão sistemática de focos potenciais adjacentes — no óstio do seio coronário, no segmento inferior da crista terminal e na válvula/crista de Eustáquio — por mapeamento de estimulação e correspondência da morfologia da onda P, o local alvo foi confirmado. A ARF ponto a ponto (40 W, 20–30 s por aplicação; irrigação de 17 mL/min) na junção VCI- AD resultou na eliminação imediata das CAPs, com atenuação acentuada dos eletrogramas locais (Figura 3). Após um período de observação de 30 minutos, a infusão repetida de isoproterenol (8 μg/min) e a estimulação programada suplementar, conforme necessário, não provocaram CAPs espontâneas nem induzidas, o que é consistente com o sucesso agudo do procedimento. Após 6 meses de acompanhamento, o paciente permaneceu assintomático e o monitoramento Holter mostrou uma redução na frequência de CAP para 3 eventos em 24 horas.

## Discussão

A maioria das CAPs se origina de regiões especializadas do miocárdio atrial com automatismo aumentado ou atividade desencadeada, incluindo as VPs (mais comuns), a veia cava superior (VCS), a crista terminal, o óstio do seio coronário e o anel tricúspide. Essas regiões são bem estudadas; por exemplo, Haïssaguerre et al. relataram em 1998 que as VPs são os gatilhos clássicos da FA,² e que critérios eletrocardiográficos e eletrofisiológicos podem auxiliar em sua localização. Por outro lado, as CAPs originadas na VCI são relativamente raras. Em um estudo com 661 pacientes com FA paroxística submetidos à ARF inicial, seis pacientes (0,91%) apresentaram FA originada na VCI.^3^ Outro estudo relatou que, entre 4.500 procedimentos de ablação de FA realizados em um único centro, apenas 2 (0,04%) envolveram FA originada na VCI.^4^ Um relato de caso recente de Wakabayashi et al. descreveu descargas ectópicas originadas do óstio da VCI em um paciente com FA paroxística, no qual as CAPs com ondas P negativas nas derivações inferiores foram mapeadas com um mapa de tempo de ativação local para um local de ativação mais precoce focal na VCI e eliminadas com sucesso por ARF.^5^

Nesse contexto, nosso caso fornece um exemplo detalhado de CAPs isoladas e altamente sintomáticas originadas da junção VCI-AD em um paciente sem FA ou taquicardia atrial documentadas e ilustra vários pontos práticos. Primeiro, a morfologia da onda P ectópica no ECG de 12 derivações sugeriu um foco no átrio direito inferior ou na VCI, levando a uma inspeção cuidadosa da junção VCI-AD em vez de atribuir as CAPs exclusivamente às veias pulmonares ou ao átrio direito inferior. Segundo, o mapeamento de ativação de alta densidade com um cateter multipolar e a reconstrução eletroanatômica tridimensional permitiram a identificação precisa da ativação mais precoce dentro da junção VCI-AD após a falha de estratégias orientadas para as veias pulmonares, evitando assim conjuntos de lesões empíricas mais extensas, como o isolamento amplo da VCI. Em terceiro lugar, um conjunto limitado de lesões de ablação focal confinadas à junção VCI-AD foi suficiente para abolir as CAPs espontâneas e induzidas por isoproterenol. Finalmente, durante o acompanhamento clínico, o paciente permaneceu livre de CAPs sintomáticas e não apresentou taquiarritmia atrial documentada.

Os mecanismos pelos quais a VCI serve como foco arrítmico provavelmente envolvem fatores estruturais e eletrofisiológicos. Um estudo inicial de Ito et al. revelou que a VCI era geralmente considerada uma zona eletricamente silenciosa, exceto por sua proximidade com a junção atrial direita.^6^ No entanto, estudos anatômicos e histológicos subsequentes demonstraram feixes de músculo estriado e fibras miocárdicas na parede anterior da VCI, indicando que esse segmento pode representar uma extensão miocárdica do átrio direito, potencialmente possuindo propriedades arrítmicas.^7,8^ Além disso, a conexão anatômica e eletrofisiológica entre a VCI e o AD pode formar um circuito de reentrada, aumentando o risco de arritmias.

Na prática clínica, a proximidade da VCI ao óstio do seio coronário e à porção inferior do átrio direito dificulta a identificação de CAPs originadas na VCI. Alguns estudos sugerem que uma onda P positiva na derivação V_1_ pode indicar um gatilho originado na veia pulmonar direita.^9^ Propomos a seguinte abordagem para suspeita de CAPs originadas na VCI: (1) Análise cuidadosa da morfologia e do eixo da onda P no ECG de superfície; ondas P positivas nas derivações inferiores sugerem focos atriais superiores, enquanto ondas P negativas sugerem focos atriais inferiores, mas a localização exata requer avaliação adicional; (2) EEF com mapeamento de ativação detalhado, com foco na porção inferior do átrio direito, óstio da VCI, anel tricúspide e óstio do seio coronário; (3) Uso de mapeamento tridimensional para confirmação anatômica; (4) do tempo e da morfologia do eletrograma intracardíaco para identificar o foco mais precoce.

No presente estudo, o ECG revelou uma onda P ectópica negativa nas derivações inferiores e uma onda P ectópica positiva na derivação V_1_, consistente com as características eletrocardiográficas de CAPs originadas na VCI. O mapeamento tridimensional localizou as CAPs na VCI durante o EEF, e a ARF foi realizada com sucesso. É importante ressaltar que a ablação na região da VCI requer cautela devido à sua complexidade anatômica e proximidade com estruturas críticas, como o nervo frênico e o SC.

Relatos anteriores de FA relacionada à VCI ressaltaram que a fluoroscopia isoladamente pode não distinguir de forma confiável a VCI de locais adjacentes, como o óstio do seio coronário ou o átrio direito inferior. Em contraste, o mapeamento eletroanatômico tridimensional permite a delimitação precisa da interface entre a veia cava inferior (VCI) e o átrio direito (AD), bem como a identificação da ativação mais precoce dentro da VCI.^10^ Em nosso caso, o mapeamento tridimensional de alta densidade foi essencial para confirmar a junção VCI-AD como a verdadeira fonte de CAPs após a falha de estratégias direcionadas às VPs, e para orientar um conjunto limitado de lesões de ablação focal em vez de uma ablação empírica mais extensa. Isso está em consonância com dados contemporâneos que apoiam a ablação personalizada e direcionada ao gatilho para focos não relacionados às VPs.

Para auxiliar os médicos na abordagem sistemática da atividade atrial ectópica originada nessa região complexa, fornecemos uma tabela comparativa que resume os padrões eletrocardiográficos, as características do eletrograma intracardíaco e as respostas à ablação associadas a locais anatômicos comuns próximos à VCI (Tabela 1). Uma maior compreensão das características eletrocardiográficas e eletrofisiológicas das CAPs relacionadas à VCI, juntamente com o uso criterioso do mapeamento tridimensional de alta densidade, provavelmente aumentará o sucesso do procedimento e melhorará os resultados para os pacientes.

**Tabela 1:** Comparação das origens de contrações atriais prematuras na região do átrio direito inferior/ veia cava inferior com rótulos e riscos harmonizados

Localização Anatômica	Características do eletrograma	Morfologia da onda-P	Resposta à ablação	Pistas Distintivas	Armadilhas/Riscos
Óstio da VCI	Ativação bipolar mais precoce na junção VCI-AD; QS unipolar no local de sucesso	Eixo inferior; frequentemente negativo em II, III, aVF com baixa amplitude	Supressão/término imediato com ablação focal no lábio VCI-AD	Atividade inicial confinada à dobradiça VCI-AD; diferenciada da crista terminal baixa, da crista de Eustáquio e do óstio do SC por mapeamento de alta densidade	Instabilidade do cateter no lábio venoso; parede fina → estouros de vapor; risco de lesão venosa se for usada potência excessiva
Anel tricúspide baixo (septal)	Sinais atriais anulares de campo próximo; podem registrar potenciais nodais His/AV de campo distante, dependendo da proximidade septal	Eixo inferior ou quase isoelétrico; polaridade precordial variável	Aplicações focais de baixa potência e curta duração são preferíveis quando se trata de radioterapia septal	Local mais precoce contíguo ao anel; o mapa de estimulação reproduz a morfologia da onda P sem captura ventricular	Proximidade ao nó AV/His (anel septal baixo) → risco de bloqueio AV; a artéria coronária direita percorre o sulco AV
Istmo cavotricuspídeo	Potenciais atriais divididos/duplos ao longo do istmo; corredor de ativação estreito entre o TA e a VCI	Frequentemente, componentes negativos no eixo inferior; podem ser sutis	Geralmente, responde bem a lesões focais ou lineares curtas no local mais precoce	A ativação mais precoce localiza-se no ICT, e não no lábio daVCI ou no óstio do SC; a ativação propaga-se ao longo do istmo	A bolsa subeustáquica causa mau contato; proximidade com a artéria coronária direita; risco de estouros de vapor no istmo fino
Óstio do SC	Sinal atrial mais precoce no SC proximal; potenciais de campo próximo nítidos no SC	Padrão bifásico ou de eixo inferior	Pode ser necessária ablação no óstio do SC ou em suas proximidades para um efeito duradouro	Localização dentro/próximo ao triângulo de Koch; ativação mais precoce dentro do SC proximal	Próximo ao nó AV e ao feixe de His; risco de bloqueio AV; potencial dissecção ou perfuração do seio coronário
Parede livre/septo do AD inferior	Potenciais atriais septais baixos ou da parede livre inferior mais precoces; sinais de campo distante da VCI podem estar presentes	Ondas negativas ou de baixa amplitude no eixo inferior	Ablação focal no eletrograma atrial mais precoce; confirmar com reexposição ao isoproterenol	O mapeamento de alta densidade separa a origem da parede livre/septal do lábio da VCI e da crista de Eustáquio	Possível compressão do nervo frênico direito ao longo do AD lateral; parede atrial fina → risco de perfuração
Crista/válvula de Eustáquio	Potenciais fragmentados ou duplos; instabilidade frequente do cateter devido à anatomia das cristas	Variável; pode mimetizar a morfologia da origem da VCI	Lesões focais precisas são frequentemente necessárias; o efeito pode ser transitório, sem estabilidade	Crista anatômica entre a VCI e o AT; o mapeamento 3D a distingue do óstio da VCI ou do SC	Contato deficiente em uma crista inclinada; estouros de vapor; ablação inadvertida perto da parede da VCI
Crista terminal baixa	Sinais atriais nítidos/fracionados; ativação ligeiramente posterior aos verdadeiros focos do lábio da VCI	Sinal negativo ou bifásico nas derivações inferiores.	Lesões focais ou lineares curtas, dependendo da extensão do envolvimento da crista	A ativação se estende ao longo da crista, em vez da dobradiça VCI-AD; diferencia-se do óstio da VCI com mapeamento denso	Captura do nervo frênico direito ao longo do AD lateral; desafios de estabilidade do cateter

AD: átrio direito; AT: anel tricúspide; VCI: veia cava inferior; VCI-AD: veia cava inferior com o átrio direito; sc: seio coronário; ICT: istmo cavotricuspídeo.

## Conclusões

Em resumo, este caso destaca a junção da VCI - AD como uma fonte incomum, porém clinicamente importante, de CAPs sintomáticas. O reconhecimento da morfologia característica da onda P e a investigação sistemática da região da VCI são cruciais, particularmente quando as CAPs persistem apesar de estratégias direcionadas às veias pulmonares ou quando se suspeita de gatilhos não relacionados às veias pulmonares. O mapeamento eletroanatômico tridimensional de alta densidade foi essencial em nosso paciente para delinear a interface venosa-atrial, confirmar a origem na VCI e guiar a ARF.

## Data Availability

Todo o conjunto de dados que dá suporte aos resultados deste estudo está disponível mediante solicitação ao autor correspondente.
