# Supplementary material for: Contrações Atriais Prematuras Originadas da Veia Cava Inferior: Uma Fonte Arritmogênica Rara Passível de Ablação
Source: Arq Bras Cardiol. 2026 May 11;123(4):e20250555. [Article in Portuguese] doi: 10.36660/abc.20250555 (PMC13398837; doi:10.36660/abc.20250555)
Supplement: Translation

# Translation: Premature Atrial Contractions Originating from the Inferior Vena Cava: A Rare Arrhythmogenic Source Amenable to Ablation

**DOI:** 10.36660/abc.20250555i

**Authors:** Ningning Zheng, Yiyuan Chen, Feng Xue, Fangfang Zhang, Lin Ling, Tingbo Jiang

**Affiliations:** 1 Suzhou City China Department of Cardiology, The First Affiliated Hospital of Soochow University, Suzhou City, Jiangsu Province – China

**Keywords:** Atrial Premature Complexes, Inferior Vena Cava, Radiofrequency Ablation

## Introduction

Premature atrial contractions (PACs) are common cardiac arrhythmias that are typically associated with trigger mechanisms. PACs originating from pulmonary veins (PVs) are considered important triggers for atrial fibrillation (AF), and pulmonary vein isolation (PVI) has been widely used as a treatment.^1^ However, the inferior vena cava (IVC) is generally regarded as an electrically quiescent region, and studies of ectopic discharges originating from the IVC are relatively rare. In this study, we present a case of PACs originating from the IVC, aiming to provide new insights into the pathophysiological mechanisms of these arrhythmias.

## Study presentation

### Patient characteristics

A 78-year-old female patient presented with recurrent palpitations, chest tightness, and fatigue over the past 9 months. Each episode lasts 10–30 minutes and occurs approximately 5–6 times per month. She denied experiencing chest pain or discomfort. Multiple electrocardiograms (ECGs) showed PACs. The patient had been treated with a β-blocker, but there was no significant improvement in symptoms, and she was subsequently referred to our hospital.

Transthoracic echocardiography (TTE) revealed mild enlargement of the left atrium, with a left atrial diameter of 40 mm. Coronary computed tomography angiography showed a noncalcified plaque in the proximal segment of the right coronary artery, accompanied by mild luminal stenosis. Other laboratory test results were within normal limits. During hospitalization, a 12-lead ECG revealed frequent ectopic PACs with negative P-wave morphology in the inferior leads (II, III, and aVF) and positive P-wave morphology in V1 (Figure 1). Holter monitoring revealed frequent PACs (15,838 events over 24 hours). The patient had no history of cardiac surgery or catheter ablation. She was diagnosed with frequent PACs and was fully informed about the status of the disease and the potential benefits and risks of various treatment options. She chose to undergo electrophysiology (EPS) and radiofrequency (RF) ablation (RFA).

**Figure 1:** Twelve-lead electrocardiogram showing premature atrial contractions with characteristic morphological features

### Ablation procedure

Preprocedural transesophageal echocardiography (TEE) confirmed the absence of left atrial appendage thrombus. Under local anesthesia, a decapolar catheter was positioned in the coronary sinus (CS), and a three-dimensional electroanatomic mapping system (CARTO 3, Biosense Webster, CA, USA) was used to reconstruct the right atrium (RA), the interatrial septum, and the caval ostia, and to perform activation mapping. High-density mapping was performed with a multipolar diagnostic catheter (Pentaray^®^ Nav Eco, Biosense Webster). RFA was delivered using an open-irrigated, contact force–sensing catheter (THERMOCOOL SMARTTOUCH SF, Biosense Webster, Diamond Bar, CA, USA) with the following settings: 40 W; irrigation 17 mL/min; lesion duration 20–30 s per application.

The EPS and the initial activation map indicated that the clinical PACs originated from the right inferior PV; therefore, PVI was performed. Despite confirmed electrical isolation of all four PVs, PACs persisted. Subsequent activation mapping localized the earliest atrial activation to the region near the fossa ovalis. Ablation at this site did not materially reduce the PACs burden.

Given the potential association between PACs and RA low-voltage areas, RA voltage mapping was performed, revealing no discrete low-voltage areas. Intravenous isoproterenol (8 μg/min) was administered to achieve a heart rate >100 bpm and provoke stable PACs, followed by remapping. High-density mapping localized the earliest activation to the posteroinferior right atrial wall near the inferior vena cava–right atrial (IVC–RA) junction, where the local atrial electrogram preceded the onset of the surface P wave by 33 ms (Figure 2).

**Figure 2:** (A) A wave at the lower posterior wall of the right atrium near the inferior vena cava orifice was preceded by 33 ms in intracardiac electrocardiography. (B), (C): Three-dimensional focal activation maps of the right atrium and the caval ostia; the red circle and arrow denote the radiofrequency ablation site at the IVC–RA junction.

Pace mapping was subsequently performed at the earliest activation site, and the paced P wave morphology closely matched the spontaneous PACs across all 12 surface ECG leads. Concurrently, the CS 9-10 bipole on the coronary sinus catheter continuously recorded PACs matching clinical morphology. After systematically excluding adjacent potential foci—at the coronary sinus ostium, the inferior segment of the crista terminalis, and the Eustachian valve/ridge—by pace-mapping and P-wave morphology matching, the target site was confirmed. Point-by-point RFA (40 W, 20–30 s per application; irrigation 17 mL/min) at the IVC–RA junction resulted in immediate elimination of PACs, with marked attenuation of local electrograms (Figure 3).

**Figure 3:** No PACs after ablation at the inferior vena cava in the intracardiac electrocardiogram.

After a 30-minute observation period, repeat isoproterenol infusion (8μg/min) and supplemental programmed stimulation as needed elicited neither spontaneous nor inducible PACs, consistent with acute procedural success.

At 6-month follow-up, the patient remained asymptomatic, and Holter monitoring showed a reduction in PAC frequency to 3 events per 24 hours.

## Discussion

Most PACs originate from specialized atrial myocardium with enhanced automaticity or triggered activity, including the PVs (most common), the superior vena cava (SVC), the crista terminalis, the coronary sinus ostium, and the tricuspid annulus. These regions are well-studied; for example, Haïssaguerre et al. reported in 1998 that the PVs are the classic triggers of AF,^2^ and that electrocardiographic and electrophysiological criteria can aid in their localization. By contrast, PACs originating from the IVC are relatively rare. In one study involving 661 patients with paroxysmal AF who underwent initial RFA, six patients (0.91%) had AF originating from the IVC.^3^ Another study reported that among 4,500 AF ablation procedures performed at a single center, only 2 (0.04%) involved AF originating from the IVC.^4^ A recent case report by Wakabayashi et al. described ectopic discharges arising from the IVC ostium in a patient with paroxysmal AF, in whom PACs with negative P waves in the inferior leads were mapped with a local activation time map to a focal earliest-activation site in the IVC and successfully eliminated by RFA.^5^

Against this background, our case provides a detailed example of isolated, highly symptomatic PACs originating from the IVC-RA junction in a patient without documented AF or atrial tachycardia and illustrates several practical points. First, the ectopic P-wave morphology on the 12-lead ECG suggested a low right atrial or IVC focus, prompting careful inspection of the IVC-RA junction rather than attributing the PACs solely to the pulmonary veins or the low right atrium. Second, high-density activation mapping with a multipolar catheter and three-dimensional electroanatomic reconstruction allowed precise identification of the earliest activation within the IVC–RA junction after pulmonary vein–oriented strategies had failed, thereby avoiding empirical, more extensive lesion sets such as wide IVC isolation. Third, a limited focal ablation lesion set confined to the IVC-RA junction was sufficient to abolish both spontaneous and isoproterenol-induced PACs. Finally, during clinical follow-up, the patient remained free of symptomatic PACs and had no documented atrial tachyarrhythmia.

The mechanisms by which the IVC serves as an arrhythmic focus likely involve both structural and electrophysiological factors. An early study by Ito et al. revealed that the IVC was generally regarded as an electrically silent zone, except for its proximity to the right atrial junction.^6^ However, subsequent anatomical and histological studies, however, demonstrated bundles of striated muscle and myocardial fibers within the anterior wall of the IVC, indicating that this segment may represent a myocardial extension of the right atrium, potentially possessing arrhythmic properties.^7,8^ Additionally, the anatomical and electrophysiological connection between the IVC and the RA may form a reentrant circuit, increasing the risk of arrhythmias.

In clinical practice, the proximity of the IVC to the coronary sinus ostium and the lower right atrium makes it challenging to identify the PACs originating from the IVC. Some studies have suggested that a positive P wave in lead V_1_ may indicate a trigger originating from the right pulmonary vein.^9^ We propose the following approach for suspected PACs from IVC: (1) Careful analysis of surface ECG P wave morphology and axis; positive P waves in the inferior leads suggest higher atrial foci, whereas negative P waves suggest lower atrial foci, but exact localization requires further assessment; (2) EPS with detailed activation mapping, focusing on low RA, IVC ostium, tricuspid annulus, and CS ostium; (3) Use of three-dimensional mapping for anatomical confirmation; (4) of intracardiac electrogram timing and morphology to identify the earliest focus.

In the present study, the ECG revealed a negative ectopic P wave in the inferior leads and a positive ectopic P wave in lead V_1_, consistent with the electrocardiographic characteristics of PACs originating from the IVC. The three-dimensional mapping localized the PACs to the IVC during the EPS, and successful RFA was performed. Importantly, ablation in the IVC region requires caution due to its anatomical complexity and proximity to critical structures, such as the phrenic nerve and CS.

Prior reports of IVC-related AF have underscored that fluoroscopy alone may not reliably distinguish the IVC from adjacent sites such as the coronary sinus ostium or the low right atrium. In contrast, three-dimensional electroanatomic mapping allows accurate delineation of the IVC–right atrium (RA) interface and identification of the earliest activation within the IVC.^10^ In our case, high-density three-dimensional mapping was essential for confirming the IVC–RA junction as the true source of PACs after PVs-oriented strategies had failed and for guiding a limited set of focal ablation lesions rather than empiric, more extensive ablation. This is in line with contemporary data supporting tailored, trigger-oriented ablation for non-PV foci.

To assist clinicians in systematically approaching ectopic atrial activity arising from this complex region, we provide a comparative table summarizing the electrocardiographic patterns, intracardiac electrogram features, and ablation responses associated with common anatomic sites near the IVC (Table 1). Greater awareness of the electrocardiographic and electrophysiological characteristics of IVC-related PACs, together with the judicious use of high-density three-dimensional mapping, is likely to enhance procedural success and improve patient outcomes.

**Table 1:** Comparison of inferior right atrial/IVC region premature atrial contractions origins with harmonized labels and risks

Anatomical Site	Electrogram Characteristics	P-wave Morphology	Response to Ablation	Distinguishing Clues	Pitfalls/Risks
IVC ostium	Earliest bipolar activation at the IVC–RA junction; unipolar QS at the successful site.	Inferior axis; often negative in II, III, aVF with low amplitude.	Immediate suppression/termination with focal ablation at the IVC–RA lip.	Earliest activity confined to the IVC–RA hinge; differentiate from the low crista terminalis, Eustachian ridge, and CS ostium by high-density mapping.	Catheter instability on the venous lip; thin wall → steam pops; risk of venous injury if excessive power is used.
Low tricuspid annulus (septal)	Near-field annular atrial signals; may record far-field His/AV nodal potentials depending on septal proximity.	Inferior axis or near-isoelectric; variable precordial polarity.	Focal low-power, short-duration applications preferred when septal.	Earliest site contiguous with the annulus; pace-map reproduces P-wave morphology without ventricular capture.	Proximity to AV node/His (low septal annulus) → risk of AV block; right coronary artery runs in the AV groove.
Cavotricuspid isthmus	Split/double atrial potentials along the isthmus; narrow activation corridor between TA and IVC.	Often inferior-axis negative components; may be subtle.	Usually amenable to focal or short linear lesions at the earliest site.	Earliest activation localizes to the CTI rather than the IVC lip or CS ostium; activation propagates along the isthmus.	Subeustachian pouch causes poor contact; proximity to the right coronary artery; risk of steam pops in thin isthmus.
CS ostium	Earliest atrial signal at proximal CS; sharp near-field CS potentials.	Biphasic or inferior-axis pattern.	May require ablation at or within the CS ostium for durable effect.	Localization within/near Koch's triangle; earliest activation within proximal CS.	Close to AV node and His; risk of AV block; potential CS dissection or perforation.
Inferior RA free wall/septum	Earliest low-septal or inferior free-wall atrial potentials; far-field signals from IVC may be present.	Inferior-axis negative or low-amplitude waves.	Focal ablation at earliest atrial EGM; confirm with isoproterenol re-challenge.	High-density mapping separates free-wall/septal origin from IVC lip and Eustachian ridge.	Possible right phrenic nerve capture along the lateral RA; thin atrial wall → perforation risk.
Eustachian ridge/valve	Fragmented or double potentials; frequent catheter instability due to ridge anatomy.	Variable; may mimic IVC-origin morphology.	Precise focal lesions often required; effect can be transient without stability.	Anatomical ridge between IVC and TA; 3D mapping distinguishes from IVC or CS ostium.	Poor contact on a sloped ridge; steam pops; inadvertent ablation near the IVC wall.
Low crista terminalis	Sharp/fractionated atrial signals; activation slightly later than true IVC lip foci.	Negative or biphasic in inferior leads.	Focal or short linear lesions, depending on the extent of the crista involvement.	Activation extends along the crista rather than the IVC–RA hinge; differentiates from IVC ostium with dense mapping.	Right phrenic nerve capture along the lateral RA; catheter stability challenges.

RA: right atrium; IVC: inferior vena cava; IVC-RA: inferior vena cava–right atrial; CS: coronary sinus; CTI: cavotricuspid isthmus; TA: tricuspid annulus.

## Conclusions

In summary, this case highlights the IVC–RA junction as an uncommon but clinically important source of symptomatic PACs. Recognition of the characteristic P-wave morphology and systematic interrogation of the IVC region are crucial, particularly when PACs persist despite pulmonary vein–oriented strategies or when non–pulmonary vein triggers are suspected. Three-dimensional high-density electroanatomic mapping was essential in our patient for delineating the venous–atrial

## Data Availability

All datasets supporting the results of this study are available upon request from the corresponding author.
